# Dietary Xenobiotics Derived from Food Processing: Association with Fecal Mutagenicity and Gut Mucosal Damage

**DOI:** 10.3390/nu14173482

**Published:** 2022-08-24

**Authors:** Sergio Ruiz-Saavedra, Aida Zapico, Carmen González del Rey, Celestino Gonzalez, Adolfo Suárez, Ylenia Díaz, Clara G. de los Reyes-Gavilán, Sonia González

**Affiliations:** 1 Department of Microbiology and Biochemistry of Dairy Products, Instituto de Productos Lácteos de Asturias (IPLA-CSIC), 33300 Villaviciosa, Spain; 2 Diet, Microbiota and Health Group, Instituto de Investigación Sanitaria del Principado de Asturias (ISPA), 33011 Oviedo, Spain; 3 Department of Functional Biology, University of Oviedo, 33006 Oviedo, Spain; 4 Anatomical Pathology Service, Central University Hospital of Asturias (HUCA), 33011 Oviedo, Spain; 5 Digestive Service, Central University Hospital of Asturias (HUCA), 33011 Oviedo, Spain; 6 Digestive Service, Carmen and Severo Ochoa Hospital, 33819 Cangas del Narcea, Spain

**Keywords:** xenobiotics, colorectal cancer, fecal mutagenicity, food processing, potential carcinogens

## Abstract

Whereas the mechanisms underlying the association of toxic dietary xenobiotics and cancer risk are not well established, it is plausible that dietary pattern may affect the colon environment by enhancing or reducing exposure to mutagens. This work aimed to investigate the association between xenobiotics intake and different stages of intestinal mucosal damage and colorectal cancer (CRC) screening and examine whether these associations may be mediated by altered intestinal mutagenicity. This was a case control study with 37 control subjects, 49 patients diagnosed with intestinal polyps, and 7 diagnosed with CRC. Lifestyle, dietary, and clinical information was registered after colonoscopy. For xenobiotics intake estimation the European Prospective Investigation into Cancer (EPIC) and the Computerized Heterocyclic Amines Resource for Research in Epidemiology of Disease (CHARRED) databases were used. The mutagenicity of fecal supernatants was assayed by the Ames test and light microscopy was used for the presence of aberrant crypt formation. Among all the potential carcinogens studied, the polyp group showed higher intakes of ethanol and dibenzo (a) anthracene (DiB(a)A). Besides, intakes between 0.75 and 1.29 µg/d of total polycyclic aromatic hydrocarbons (PAHs) were related with a higher risk of belonging to the polyp group. On the contrary, an intake of wholegrain cereals greater than 50 g/d was associated with a reduction in the relative risk of belonging to the polyp group. Heterocyclic amines (HAs) such as 2-amino-1-methyl-6-phenylimidazo (4,5,b) pyridine (PhIP) were associated with an increased level of mutagenicity in polyps. This study is of great interest for the identification of possible therapeutic targets for the early prevention of colon cancer through diet.

## 1. Introduction

Despite the progress that has been achieved in the early detection of colorectal cancer (CRC) in the last few years, this disease is one of the most frequently diagnosed and the second leading cause of death in Spain [1,2]. In addition to the genetic factors, age, or the presence of colon polyps, several epidemiological studies have also identified lifestyle factors either promoting or protecting against CRC. Of them, obesity, smoking habit, alcohol consumption, and diet are accepted risk factors for this pathology [3,4,5]. Through foods, humans are exposed to complex mixtures of substances that may cause, modulate, or prevent diseases. However, there is not enough conclusive scientific evidence on the effect of dietary habits on the development of CRC. From all food groups, red and processed meats are considered the most scientifically proven CRC risk factors, being classified by the International Agency for Research on Cancer (IARC) as “carcinogenic” and “probably carcinogenic” to humans, respectively. In addition, diets with high content of sugar, animal products, and alcohol have been related to CRC development, contrary to whole grains, fruits, and vegetables, which have shown a protective effect [3]. In the general population, diet represents one of the major factors of exposure of the colonic epithelium to mutagenic and genotoxic compounds, being involved in both the initiation of cell transformation and tumor progression [6,7]. Nitrosamines (NA) (formed during the preservation process applied to some types of foods), heterocyclic amines (HAs), (derived from creatinine, amino acids, and sugars), and polycyclic aromatic hydrocarbons (PAHs) (formed from the incomplete combustion of organic compounds), are the major mutagenic/genotoxic compounds derived from food processing and have accumulated strong scientific evidence of their relationship with cancer in animal studies [8,9,10,11]. Among PAHs, a positive association has been observed between the intake of benzo(a)pyrene (B(a)P) through the consumption of meat mainly cooked on the grill or barbecue (daily intakes ≥ 2.7 ng/d) and the probability of developing rectal adenomas [12]. Regarding HAs, some carbolines have a mutagenicity index more than 1000 times higher than that of hydrocarbons such as B(a)P, which evidences their potential toxicity [13]. Despite some food components having genotoxic potential, others, such as some bioactive compounds derived from vegetable foodstuffs, have shown to inhibit various stages of the carcinogenic process [14,15,16,17]. For example, it has been shown that the intake of nitrates over 142.5 mg/d can increase the risk of CRC only when the daily intake of vitamin C is under 83.9 mg/d [18]. On the other hand, the intake of nitroso-dimethylamine (NDMA) ≥ 0.07 μg/d was associated with an increased risk of this pathology when daily doses of vitamin E were under recommendations [19]. In this regard, fiber consumption may have the potential to decrease CRC risk by means of increasing the colonic transit rate, diminishing the exposure of colonic epithelial cells to ingested carcinogens, or by promoting proliferation of some beneficial microorganisms such as some colonic butyrate producers [20,21,22,23]. Most dietary sources of fiber are also known to contain phenolic compounds such as flavonoids, phenolic acids, anthocyanins, or lignans, which have received extensive attention because of their chemoprotective actions in animal models and human epidemiology studies [24].

The process of progressive intestinal mucosa damage leading to CRC can take several years. Polypous endoscopic lesions, frequently accompanied by histological examination, are used in routine clinical practice to determine the intestinal mucosal damage and CRC stage. Hyperplastic polyps present low risk of evolving to neoplasia, whereas serrated (traditional and sessile serrated adenomas) and adenomatous (tubular, tubulovillous, villous) polyps, with a low or high grade of dysplasia, present a progressively augmented risk of adenocarcinoma development [25]. One of the earliest events in CRC is the formation of aberrant crypt foci (ACF) in the colonic mucosa (normal or typical without cell alterations or with hyperplasia or dysplasia), which occurs when the process is still reversible. However, the analysis of ACF is not a routine practice in the diagnosis and prognosis of CRC [26].

Scarce information is still available about the biological relevance of some dietary components produced during food processing or cooking on the mutagenicity and genotoxicity of the intestinal environment. Although the mechanisms to explain the relationship between toxic xenobiotics in diet and cancer risk are not well established yet, it is plausible that differences in the dietary habits affect the colonic environment by increasing or reducing the exposure to mutagens. Therefore, the aim of this work was to analyze the impact of xenobiotics intake as related to different stages of intestinal mucosa damage and CRC and to examine whether these associations may be mediated through modification of intestinal mutagenicity.

## 2. Materials and Methods

### 2.1. Study Design and Volunteers

This transversal analysis is part of the broader project “Effect of Diet and exposure to XEnobiotics generated during food processing on the genotoxic/cytotoxic capacity of the intestinal Microbiota” (MIXED).

**Figure 1:**
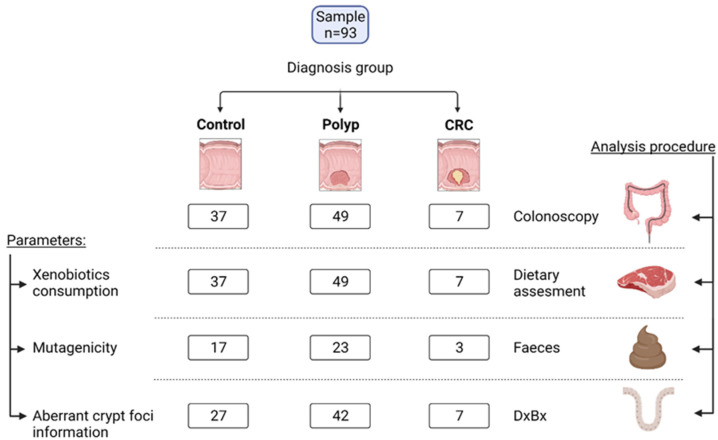
Graphical information on the study design and sample size. The left column information indicates the variables studied and the right column text indicates the methodology or the raw material used to study those variables. Each box in the central part of the figure shows the number of volunteers belonging to the different diagnosis group, with available data for each of the determinations. CRC, colorectal cancer; DxBx, pathological analysis.

The recruitment of volunteers and collection of human samples (faeces and biopsies of intestinal mucosa), anamnesis, and analytical controls of volunteers was carried out from October 2019 to December 2021 by the faculties of the Digestive Section from the Central University Hospital of Asturias (HUCA) and the Carmen and Severo Ochoa Hospital from Asturias, in the north of Spain. Volunteers were selected among patients who came to the hospital for consultation due to clinical symptoms or from those included in the colon cancer screening program in our region. Three groups of control patients (n = 37), patients diagnosed with intestinal polyps (n = 49), and patients diagnosed with CRC (n = 7) were recruited among adults submitted to a diagnostic colonoscopy (Figure 1). The following exclusion criteria were applied: age <40 or >75 years, treatment with omeprazole, antibiotics, corticoids, non-steroidal anti-inflammatory drugs, or specific cancer treatment at the time of the study or in the previous two months, previous surgery of the digestive system, autoimmunity, altered thyroid function, or history of diabetes or goiter. Patients were asked to provide a stool sample collected prior to the preparation for colonoscopy. A biopsy of intestinal mucosa was extracted during colonoscopy for examination of ACF at the Pathological Anatomy Section at HUCA.

Those individuals interested in participating were informed of the objectives of the study and signed an informed consent form. This project was evaluated and approved by the Regional Ethics Committee of Clinical Research of Asturias (Ref. 163/19) and by the Committee on Bioethics of CSIC (Ref. 174/2020). The procedures were performed in accordance with the fundamental principles set out in the Declaration of Helsinki, the Oviedo Bioethics Convention, and the Council of Europe Convention on Human Rights and Biomedicine, as well as in Spanish legislation on bioethics. Directive 95/46/EC of the European Parliament and the Council of October 1995, on the protection of individuals regarding the processing of personal data and on the free movement of such data, was strictly followed.

### 2.2. Nutritional Assessment

Dietary information was obtained from patients when they arrived for colonoscopy results at the medical consultation by means of a personalized interview conducted by trained interviewers. Exceptionally, as a result of the pandemic and COVID-19 restriction of visitors to hospitals in Spain, some of the surveys were conducted through online tools. For this purpose, a semi-quantitative food-frequency questionnaire (FFQ) was constructed with 155 items. In addition to food and culinary preparations, the specific type of food was recorded, as well as cooking methods and other related questions, when necessary. For each food, the frequency of intake and portion size were registered by means of a validated photograph album adapted from the Pilot Study for Assessment of Nutrient Intake and Food Consumption Among Kids in Europe (PANCAKE) [27]. A specific section about cooking habits (boiled, fried, grilled, baked/broiled, or barbecued) and the degree of cooking or toasting in the case of meats, fried potatoes, or toasted bread (undercooked, medium, well done, very well done) was included in the FFQ. To standardize this point, photographs of the different temperatures, in which the degree of browning increased progressively, were developed specifically for this study: low, medium, well done, and very well done were incorporated. Additionally, complementary questions such as which part of the food was consumed (breast or thigh in the case of chicken) or the possible consumption and/or cooking of the skin (cooking with skin and eating the skin; cooking with skin but not consuming it; and cooking without skin) were incorporated in order to improve the quality of the information. The intake of xenobiotics obtained from FFQ was previously validated by means of a 24 h food dietary recall [28].

The classification of the food into food groups was carried out according to the Centre for Higher Education in Nutrition and Dietetics (CESNID) criteria [29]. Food composition tables of CESNID [29] and the United States Department of Agriculture (USDA) [30] were used to transform food consumption into energy and macronutrient intake. The phenolic content of the foods was extracted from Phenol Explorer 3.6 [31] and fiber content from the tables by Marlett and Cheung [32].

### 2.3. Xenobiotics Derived from Food Processing

Based on food consumption per individual, cooking method, cooking time, and degree of browning, the nutritional analysis of the sample was carried out. For this purpose, information on the consumption of HAs, PAHs, nitrates, and nitrites was obtained mainly from the European Prospective Investigation into Cancer and Nutrition (EPIC) carcinogen database [33]. The EPIC database compiles information obtained from 139 references regarding the content per 100 g of food in nitrosamines, HAs, PAHs, nitrites, and nitrates in more than 200 food items. The food composition table is classified according to the preservation method, cooking method, degree of browning, and temperature [33]. HA and B(a)P information was completed with the Computerized Heterocyclic Amines Resource for Research in Epidemiology of Disease (CHARRED) [34], whereas in the case of nitrates European Food Safety Authority (EFSA) information was used [35]. Acrylamide content was provided by the U.S. Food and Drug Administration (FDA) composition tables [36] and other external reference sources were used for acrylamide [37,38,39], HAs [40], total PAHs [41], and nitrosamines [42,43,44,45].

### 2.4. Anthropometrical Determinations 

Height (m) and weight (kg) were taken by standardized protocols [46]. Body mass index (BMI) was calculated using the formula weight/(height)^2^. Subjects were classified into normal weight (18.5–24.9 kg/m^2^), overweight (25.0–29.9 kg/m^2^), and obese (≥30.0 kg/m^2^), based on the Spanish Society for the Study of Obesity (SEEDO) criteria [47]. 

### 2.5. Pathological Assessment

A total of 76 biopsies of colorectal mucosa fixed with 10% formaldehyde and paraffin-embedded were analyzed. Serial tissue sections were stained with hematoxylin–eosin and analyzed by light microscopy for the presence of ACF. Discordant diagnoses were reviewed to reach a consensus. Based on previously reported categorizations [48,49,50,51] histological findings were classified into 3 groups: normal–typical ACF (crypt with increased diameter only), hyperplastic ACF, and dysplastic ACF.

### 2.6. Fecal Samples and Mutagenicity

Fecal samples were collected in sterile plastic containers at hospitals participating in the study. Samples were frozen after deposition within a period not exceeding two hours and transported to the laboratory. Four grams of frozen samples were weighted, diluted 1/10, and homogenized with sterile PBS in a LabBlender 400 Stomacher (Seward Medical, London, UK) for 3 min at maximum speed. Samples were centrifuged for 15 min at 4 °C and 14,000 rpm and the obtained supernatants were separated from pellets and kept frozen at −20 °C until use. 

The mutagenicity of fecal supernatants was assayed by the Ames test, without metabolic activation, against the strain *Salmonella enterica* serovar typhimurium TA100 using the 5051 Muta-ChromoPlate^TM^ kit (EBPI, Mississauga, ON, USA) and following the manufacturer’s instructions, with minor modifications. Briefly, fecal supernatants were thawed on ice, filtered through Amicon^®^ Ultracel 3K filters (Merck Milipore Ltd., Cork, Ireland) at 16,000× *g* and 4 °C for 30 min, and serially diluted with sterile mili-Q water at 1/150-1/200-1/250, or 1/300-1/350-1/400. Fecal supernatant dilutions were combined with S. *typhimurium* TA 100 strain grown over 16 h at 37 °C in the sterile liquid medium provided by the manufacturer and the solution mix containing Davis–Mingoli salts, D-glucose, bromocresol purple, D-biotine, and L-histidine in the concentrations indicated by the manufacturer. Positive control (including sodium azide as a mutagen, grown bacteria, and solution mix), negative control (including only solution mix), and the appropriate series of dilutions of fecal supernatants (providing a variable number of positive revertant wells) were each added to 96-well microtiter plates containing 200 µL per well and incubated at 37 °C for 5 days. Reversion rates (RR) in fecal dilutions were calculated for conditions displaying the following criteria: less than 96 revertant wells per plate and more than 48 revertant wells in positive control. Considering the dilution factor, the level of mutagenicity was expressed as the mean of values corresponding to the three dilutions tested for each sample. 

Given that fecal mutagenicity in our assays was determined by the frequency of reversion of the L-histidine auxotrophy present in the strain *S. Typhimurium* TA100, we first ruled out any interference of this amino acid, naturally present in fecal samples, with the mutagenicity assays performed. To this end, L-histidine levels of fecal supernatant dilutions tested were determined by ultra-high-performance liquid chromatography (UHPLC) using the method described by Redruello et al. [52] and adapted to fecal supernatants by Salazar et al. [53]. The highest level of histidine among our fecal dilutions was 2.53 µM, the rest of samples being below this value. Then, the mutagenicity of serial increasing concentrations of aqueous dilutions of L-histidine (0.5, 1.0, 1.5, 2.0, 2.5, 3.0, 3.5, 4.0, and 5 µM) was tested following the same procedure as indicated above. The Spearman correlation coefficient (see 4.7) for aqueous L-histidine concentrations and RR values in the mutagenicity reversion test (0.5833, 2.125, 2.5833, 1.75, 2.1666, 1.6666, 1.9166, 1.9166, 2.3333) was 0.226 (*p*-value = 0.559; not significant), allowing us to discard any interference of L-histidine with the mutagenicity assays of our fecal supernatants in the conditions described here.

### 2.7. Statistical Analyses 

Results were analyzed using the IBM SPSS software version 25.0 (IBM SPSS, Inc., Chicago, IL, USA) and RStudio software version 1.4.3. Goodness of fit to the normal distribution was checked by means of the Kolmogorov–Smirnov test. As normality of the variables was not achieved, nonparametric tests were used. Overall, categorical variables were summarized as percentages and continuous ones as median and interquartile range (IQR = Q1–Q3). Fisher and Z tests and Kruskal–Wallis and Mann-Whitney U tests were performed for categorical and continuous variables, respectively (*p*-value < 0.05), with Bonferroni correction. Logistic regressions were calculated through categorical tertiles of consumption of each variable and adjusted by age and BMI. To more deeply explore the associations between fecal mutagenicity and dietary components, Spearman correlation analyses were conducted. A heatmap was generated using the RStudio software version 1.4.1103 package corrplot. GraphPad Prism 9 was used for graphical representations. 

## 3. Results

General characteristics, anthropometric parameters, factors related to intestinal function, and anatomopathological diagnosis are presented in Table 1 for the three clinical endoscopic diagnosis groups: healthy controls, polyps, and CRC. In spite of no significant differences being found in the gender ratio between the polyp group and controls, CRC patients were exclusively males. Individuals in the polyp group had higher BMI and lower physical activity than controls. 

**Figure 2:**
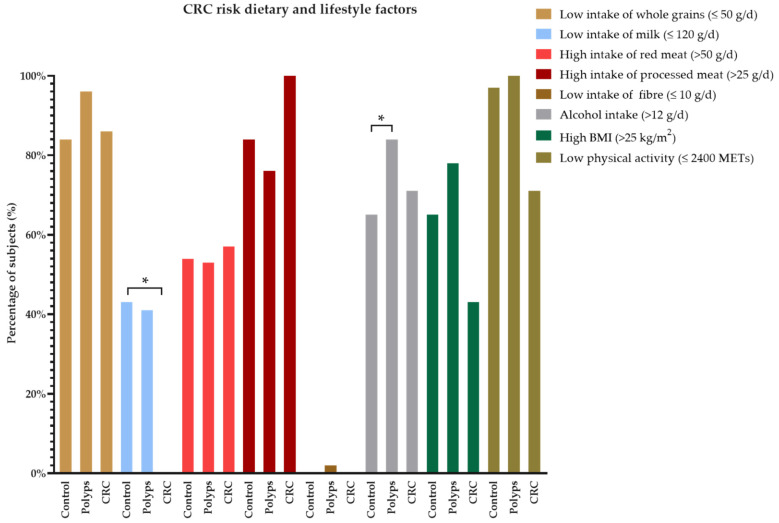
Analysis of the dietary and lifestyle significant risk factors for CRC according to GBD and clinical diagnosis group. The control group is used for comparison. BMI, body mass index; CRC, colorectal cancer; GBD, Global Burden of Diseases, Injuries, and Risk Factors Study; MET, metabolic equivalent of task. (*) *p*-value < 0.05.

Variations in the dietary intake and lifestyle factors related to CRC risk, according to the Global Burden of Diseases, Injuries, and Risk Factors Study (GBD) [54], in polyp and CRC groups vs. control are presented in Figure 2. According to the results, the percentage of individuals in the polyp group consuming alcoholic beverages (>12 g/d) was higher than in the control (84% vs. 65%; *p*-value < 0.05). Differences were also found for the percentage of CRC patients consuming milk under 120 g/d when compared to the control group (0% vs. 43%). Although not significant, the CRC group showed the highest consumption of red and processed meats (>50 and >25 g/d, respectively).

On the other hand, the median intake of bioactive and potential carcinogenic compounds (xenobiotics and ethanol) in the total sample and polyp and CRC groups vs. control is presented in Table 2. The intake of ethanol and DiB(a)A was significantly increased in the polyps group compared to the control (8.13 vs. 1.88 and 0.07 vs. 0.04, respectively). 

In order to evaluate the overall impact of GBD risk-related factors along with the intake of bioactive and carcinogenic compounds into the polyp risk, logistic regression analyses adjusted by age and BMI were conducted (Table 3). According to the results obtained, there was a higher risk of belonging to the polyps group for those subjects with an alcohol consumption greater than 48 g/d. The consumption of more than 60 g/d increased the risk of polyps by 3 (OR = 3.01; *p*-value = 0.020; data not shown). Regarding protective factors, the consumption of more than 50 g/d of whole grains led to an 83% decrease in the risk of being in the polyp group. On the other hand, xenobiotics such as Total PAH were associated with a three-fold increase in the risk of polyps.

**Figure 3:**
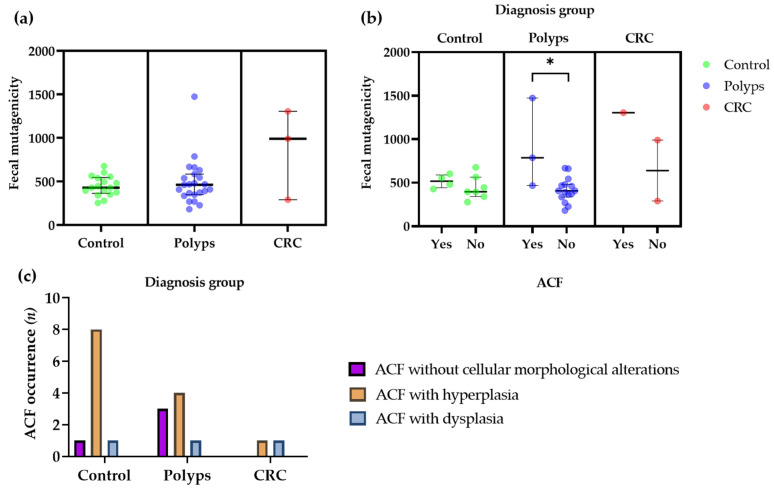
Dot plots comparing the mutagenicity of volunteers’ fecal samples according to (**a**) the clinical endoscopic diagnosis group and (**b**) the ACF occurrence. The fecal mutagenicity for each volunteer is represented by colored circles. Wide horizontal lines indicate the median for each condition and error bars represent the interquartile range or the range for the CRC group. (*) Significant differences between groups (*p* ≤ 0.05). (**c**) ACF occurrence and type (hyperplastic, dysplastic, or without cellular morphological alterations) in the sample for each clinical endoscopic diagnosis group (control, polyps, and CRC). Each bar represents the number of cases detected for each ACF category in each diagnosis group. ACF, aberrant crypt foci; CRC, colorectal cancer.

**Figure 4:**
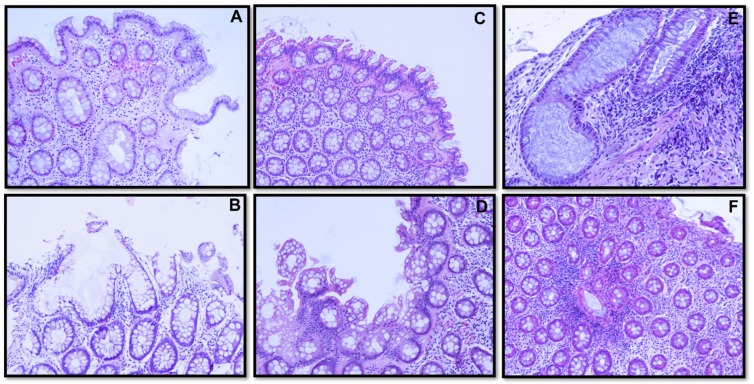
Photographs of histological sections showing ACF (H&E stain). Non-dysplasic distorted architecture (cells without morphological alterations) ((**A**,**B**), ×100). Hyperplastic with “serrated” lumen, “sawtooth” appearance ((**C**,**D**), ×100). Low-grade dysplasia with enlarged crypts, nuclear stratification, mucin depletion, elongated and hyperchromatic nuclei with loss of nuclear polarity and associated lymphocytic infiltrate ((**E**), ×200; (**F**), ×100). ACF, aberrant crypt foci; H&E, hematoxylin and eosin stain.

Differences in fecal mutagenicity levels according to the clinical diagnosis group are presented in Figure 3a, with no significant differences found among groups. A significant increase in fecal mutagenicity was observed for those individuals from the polyp group presenting ACF (Figure 3b), and a trend to higher fecal mutagenicity, not reaching statistical significance, was also obtained for individuals presenting ACF in the other two groups (control and CRC) (Figure 3b). A higher occurrence of ACF with hyperplasia was observed in control and polyp groups compared to other types of ACF (with dysplasia or without cell alterations) (Figure 3c). In the CRC group, two patients presented ACF, either hyperplastic or dysplastic. The occurrence of ACF in intestinal mucosal samples was analyzed through histomorphological evaluation of colorectal mucosa sections. Two samples of normal (without cellular morphological alterations) (Figure 4A,B), hyperplastic (Figure 4C,D), and dysplastic (Figure 4E,F) ACF from different subjects are shown in Figure 4.

**Figure 5:**
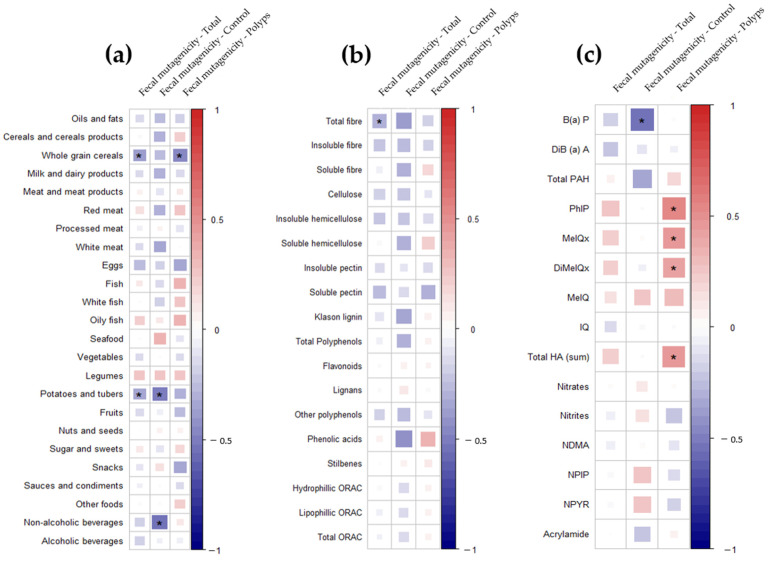
Heatmap defined by Spearman correlations between fecal mutagenicity and (**a**) food groups, (**b**) bioactive compounds, and (**c**) xenobiotics. Blue and red colors represent negative and positive associations, respectively. The color intensity is proportional to the degree of association between fecal mutagenicity and the factors considered. (*) *p* ≤ 0.05.

**Figure 6:**
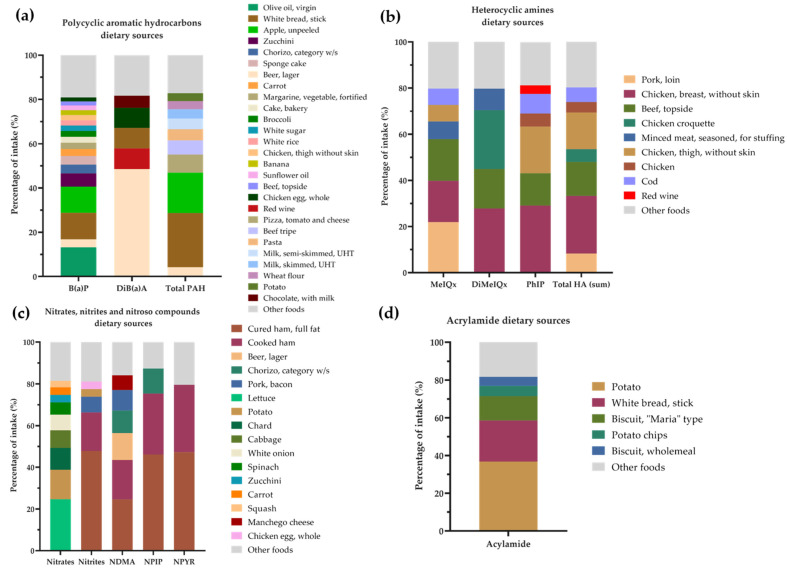
Major dietary sources of (**a**) polycyclic aromatic hydrocarbons; (**b**) heterocyclic amines; (**c**) nitrates, nitrites, and nitroso compounds; and (**d**) acrylamide in the sample. Only the most frequently consumed xenobiotics were considered. For each compound, food items accounting for at least 80% of total intake were included. B(a)P, benzo (a) pyrene; DiB(a)A, dibenzo (a) anthracene; DiMeIQx, 2-amino-3,4,8 trimethylimidazo (4,5,f) quinoxaline; MeIQx, 2-amino-3,8 dimethylimidazo (4,5,f) quinoxaline; NDMA, N-nitrosodimethylamine; NPIP, N-nitrosopiperidine; NPYR, N-nitrosopyrrolidine; PhIP, 2-amino-1-methyl-6-phenylimidazo (4,5,b) pyridine; Total HAs, total heterocyclic amines; Total PAHs, total polycyclic aromatic hydrocarbons.

Correlations between the intake of the major food groups, dietary bioactive compounds (fibres and polyphenols), and dietary xenobiotics with fecal mutagenicity are presented in Figure 5. Intake of whole grains, soft drinks, potatoes, and tubers, as well as fiber and B(a)P intake, showed an inverse association with mutagenicity levels (Figure 5a–c). In contrast, in the polyp group, PhIP, MeIQ, MeIQx, DiMeIQx, and total HA were positively associated with the mutagenicity (Figure 5c). Mutagenicity mean levels were also higher for those individuals presenting higher BMI (>25 kg/m^2^) (515.97 vs. 464.74, *p*-value < 0.05; data not shown). These differences were persistant for indivuals belonging to the control and polyp groups. We finally analyzed the main dietary sources of xenobiotics such as Total PAH and DiB(a)A, the carcinogens that, along with ethanol, displayed the highest risk for polyps in our study population (Figure 6a). Alcoholic beverages such as beer and wine were the main sources of DiB(a)A intake in the sample, whereas meats such as chicken, beef, and pork loin contributed to explaining almost 80% of the total HA intake (Figure 6b). Indeed, total HA values were greatly influenced by PhIP consumption from chicken breast (29% from total xenobiotics consumption), DiMeIQx from chicken breast (28%) and croquettes (26%), and MeIQx from pork loin (22%) and beef (18%). The processed meats of cured and cooked ham were the main dietary sources of nitrites (48% and 18%, respectively) and nitrosamines (Figure 6c), whereas nitrates were derived mainly from vegetables (Figure 6c) and acrylamide from cereal-derived products (potato, white bread, and cookies) (Figure 6d). 

## 4. Discussion

There are controversial findings in the literature about the impact of environmental factors on the risk of CRC. It is generally assumed that diets with a high content of animal fat, alcohol, and processed meat and low in milk products, calcium, and whole grains increase the risk of CRCs, whereas those with a high presence of fruit and vegetables reduce it [55,56,57,58]. Thus, the detection in our study of a higher consumption of alcoholic beverages and lower consumption of whole grains on the risk of polyps support these hypotheses. Though cancer–food relationships appear to be very complex, several food components may be potentially genotoxic [59]. Given the scarce knowledge in the literature about the interactions between the different food-related genotoxic and protective factors, the observation of changes in mutagenicity levels associated with the dietary balance between the intake of substances with a possible pro-carcinogenic effect, such as dietary xenobiotics and the consumption of potentially beneficial components such as dietary fibers, may be the main novel contributions of this research work.

The intake of xenobiotics in our study sample was similar to that reported by other authors applying comparable methodology [28,60]. In this line, the intake of total HA in the sample (especially DiMeIQx, MeIQx, and PhIP) fell into the range of the third quartile of intake of an EPIC study [61]. In addition, the intake of hydrocarbons such as B(a)P, DiB(a)A, and total PAHs showed similar values as previously described in Spain [28,40]. Regarding dietary sources, in general terms they were in consonance with other European countries. HA derived mainly from meats such as chicken, beef, and pork, whereas processed meats were the main dietary sources of nitrosamines and nitrites [28]. Carbohydrate-rich foods such as potato and bread were identified as the main dietary sources of acrylamide [28,62]. Finally, in our sample, PAH was the xenobiotics group with the highest variety of dietary sources, especially in the case of B(a)P, which mainly derived from vegetable foodstuffs such as olive oil, white bread, apple, and zucchini. Similar to other works, total PAHs were mainly provided by cereals and oils and fats [40,63], whereas the alcoholic beverage of lager beer was the major DiB(a)A foodstuff according to other studies [28]. 

Based on our results, we are not able to propose the existence of a dietary pattern associated with the presence of polyps or CRC. In line with several previous studies, a significant increase in the consumption of alcoholic drinks was observed in the polyp group respective to the controls [64,65]. In this regard, alcoholic beverage consumption in the sample, particularly beer, cider, and red wine, was around five times higher in the polyp group (data not shown). Elucidating the possible mechanisms behind this association, the eternal question is whether the observed effects are attributable to the ethanol content of these beverages or whether they could be protective based on their content in phenolic compounds. Although given the nature of the study we cannot establish causality, our data suggest that ethanol consumption above 12 g/d increases by 2.7 times the risk of being in the polyp group (*p*-value 0.05; data not shown). Consistent with this finding, the intake of stilbenes, mainly derived from red wine, was higher in the group of individuals with polyps, although the differences did not reach statistical significance. It may be of interest that although some meta-analyses have reported a 17% additional risk of CRC per 100 g/d of red meat consumption, we did not find this increased risk [66]. A possible explanation may be that even when the intake of meat and meat products in our study was within the range of the average consumption of America, Australia, New Zealand, Europe, and Russia [67], the proportion of red meat was considerably lower with respect to these other populations. In contrast to countries such as America, where red meat represents around 50% of the total meat consumed, in the present study the proportion was around 30% for both healthy individuals and those with polyps (data not shown). According to previous epidemiological evidence, in this work it was found that wholegrain cereals consumption greater than 50 g/d was associated with a reduction in the relative risk of belonging to the polyp group [68,69]. Several hypotheses have been put forward to explain the mechanisms that may connect whole grains to the risk of CRC. It is plausible that the high fiber content of these foods, together with the presence of several dietary compounds with antioxidant activity, may be some of the underlying causes for this connection. Certain components of cereal fiber exhibit some physiological effects, with a potential positive impact on CRC, including the ability to shorten intestinal transit time and the modulation of intestinal lipid and glucose absorption. In addition, some studies have highlighted the protective effect of high-fiber dietary patterns at the gut level by modulating the composition and activity of the intestinal microbiota [70,71].

The development of CRC is a long-term process in which a complex series of changes take place, culminating in the formation of a carcinoma. During this process, a healthy mucous membrane can suffer morphological transformations resulting in ACF formation and hyperplasia [72,73]. Due to the epidemiological and genetic association of ACF with initial intestinal lesions, they may be suggested as CRC biomarkers [26]. Although there is limited research on this subject, a greater ACF occurrence has been observed in people with BMI > 35 kg/m^2^ and in those with a diet with a high intake of meat and low residues content [74,75]. In this complex scenario, we propose that an alteration in the balance between compounds with pro-carcinogenic activity and those with a protective role could favor the generation of a mutagenic environment at the colonic level, promoting the appearance of ACF. To test this hypothesis, the intake of food groups, xenobiotics, and bioactive compounds was related to fecal mutagenicity levels through Spearman correlations. Total HA, DiMeIQx, MeIQx, and PhIP were positively associated with increased fecal mutagenicity, contrary to whole grains and other xenobiotics such as B(a)P (probably due to its mostly vegetable-derived dietary sources). Indeed, greater mean mutagenicity levels were found for higher BMI values. Furthermore, we also observed that the risk of developing a lesion identifying a precancerous stage was significantly higher in those subjects with polyps who had a higher dietary intake of pro-carcinogenic compounds such as ethanol. This is of great interest for the identification of possible therapeutic targets for the prevention of early colon cancer through diet. 

Limitations of the study: Although relationships between diet and risk of polyp group membership were observed, it was not possible to establish causality in this association. Despite the high degree of detail carried out in the recording of dietary information, including the efforts made to quantify the degree of cooking of foods, this information is difficult to quantify accurately. It would be desirable in the future to use biological markers to validate the accuracy of the collection of dietary information.

## 5. Conclusions

This preliminary study points to ethanol and dibenzo(a)anthracene (DiB(a)A) intake as potential factors related to the increase in intestinal polyp risk and to the intake of whole-grain cereals above 50 g/d as a protecting factor. The intake of some of the heterocyclic amines under evaluation, such as 2-amino-1-methyl-6-phenylimidazo(4,5,b)pyridine (PhIP), was associated with a higher level of fecal mutagenicity in the polyp group. This study is of great interest for the generation of new hypotheses focused on designing nutritional strategies for early prevention of colon cancer.

## Figures and Tables

**Table 1:** General description of the sample population according to diagnosis group.

	Control (*n* = 37)	Polyps (*n* = 49)	CRC (*n* = 7)
Male gender	17 (45.95) _a_	30 (61.22) _a_	7 (100.00) _b_
Age (years)	60 (54–66) _a_	63 (56–66) _a_	63 (61–70) _a_
Energy intake (kcal/d)	1974.87 (1492.99–2463.87) _a_	1926.43 (1691.48–2675.80) _a_	2070.43 (1915.76–2830.29) _a_
BMI (kg/m^2^)	25.70 (23.67–28.94) _a_	27.56 (25.14–31.22) _b_	24.68 (24.21–29.32) _a_
CRC history (1st grade)	9 (24.32) _a_	11 (22.45) _a_	1 (14.29) _a_
Physical activity (min/d)	75.00 (37.50–75.00) _a_	50.00 (37.50–75.00) _a_	90.00 (75.00–90.00) _b_
Sleeping (hours/d)	7.00 (6.00–7.25) _a_	7.00 (6.00–8.00) _a_	7.00 (6.00–8.00) _a_
Current smoker	6 (16.22) _a_	13 (26.53) _a_	1 (14.29) _a_
Gastrointestinal functionality			
Deposition/week	8.50 (6.00–8.50) _a_	7.00 (6.00–8.50) _a_	8.50 (8.50–8.50) _a_
Liquid feces	0 (0.00) _a_	1 (2.04) _a_	-
Soft feces	27 (72.97) _a_	32 (65.31) _a_	5 (71.43) _a_
Hard feces	10 (27.03) _a_	16 (32.65) _a_	2 (28.57) _a_
Pathological analysis (DxBx)			
HP	4 (10.81) _a_	7 (14.29) _a_	0 (0.00) _a_
TA	0 (0.00) _a_	22 (44.90) _b_	-
TVA	0 (0.00) _a_	5 (10.20) _b_	-
SSA	0 (0.00) _a_	1 (2.04) _a_	-
HGD	0 (0.00) _a_	6 (12.24) _b_	-
AC	0 (0.00) _a_	1 (2.04) _a_	7 (100.00) _b_
LSC	24 (64.86) _a_	3 (6.12) _b_	0 (0.00) _b_
Not available	9 (24.32) _a_	4 (8.16) _b_	0 (0.00) _a_

Values are presented as median (IQR = interquartile range = Q1–Q3) for continuous variables or number (%) for categorical ones. Values in the same row showing different subscripts present a statistically significant difference (*p* ≤ 0.05) than control group. BMI, body mass index; CRC, colorectal cancer; DxBx, pathological analysis; HP, hyperplastic polyps; TA, tubular adenoma; TVA, tubulovillous adenoma; SSA, sessile serrated adenoma; HGD, high-grade dysplasia; AC, adenocarcinoma; LSC, less severe conditions.

**Table 2:** Differences in the median intake of bioactive and carcinogenic compounds in the total sample and by diagnosis group.

Variables	Total Sample (*n* = 93)	Diagnosis Group		
		Control (n = 37)	Polyps (n = 49)	CRC (n = 7)
Bioactive				
Total fiber (g/d)	20.88 (14.77–25.15)	21.89 (14.77–26.87) _a_	20.41 (15.22–23.75) _a_	22.33 (13.80–29.55) _a_
Insoluble fiber (g/d)	12.50 (8.66–15.08)	12.50 (8.66–16.57) _a_	12.29 (9.25–14.64) _a_	13.27 (8.04–17.70) _a_
Soluble fiber (g/d)	2.40 (1.87–3.06)	2.62 (1.86–3.17) _a_	2.32 (1.88–2.85) _a_	2.77 (1.91–3.03) _a_
Cellulose (g/d)	5.01 (3.62–6.39)	5.01 (3.50–6.40) _a_	4.93 (3.64–6.24) _a_	5.35 (2.97–8.02) _a_
Insoluble hemicellulose (g/d)	3.88 (2.80–4.90)	4.02 (2.86–5.38) _a_	3.63 (2.68–4.51) _a_	4.01 (2.69–5.48) _a_
Soluble hemicellulose (g/d)	1.65 (1.15–2.27)	1.77 (1.09–2.32) _a_	1.57 (1.19–2.04) _a_	1.90 (1.04–2.35) _a_
Insoluble pectin (g/d)	1.34 (1.01–1.96)	1.53 (1.11–2.02) _a_	1.29 (0.94–1.81) _a_	1.58 (1.20–2.06) _a_
Soluble pectin (g/d)	0.66 (0.51–0.89)	0.70 (0.55–0.92) _a_	0.62 (0.45–0.88) _a_	0.69 (0.58–1.29) _a_
Klason lignin (g/d)	1.63 (1.22–2.26)	1.63 (1.30–2.26) _a_	1.69 (1.22–2.11) _a_	1.42 (1.13–2.44) _a_
Total polyphenols (mg/d)	1482.46 (963.00–1951.48)	1509.00 (1074.04–1877.40) _a_	1376.33 (904.37–1951.48) _a_	1454.97 (1000.45–2051.10) _a_
Flavonoids (mg/d)	128.09 (72.89–302.17)	136.93 (78.28–251.34) _a_	122.87 (53.65–331.58) _a_	174.05 (80.98–498.80) _a_
Lignans (mg/d)	46.95 (26.29–74.22)	47.39 (28.61–85.26) _a_	40.18 (22.28–60.34) _a_	55.39 (30.43–92.01) _a_
Other polyphenols (mg/d)	24.27 (15.19–42.26)	27.32 (16.37–45.15) _a_	19.93 (14.86–35.08) _a_	29.48 (19.32–60.48) _a_
Phenolic acids (mg/d)	496.08 (211.63–836.53)	609.92 (222.81–958.37) _a_	386.62 (188.52–781.00) _a_	496.08 (262.91–1223.65) _a_
Stilbenes (mg/d)	0.11 (0.04–0.76)	0.09 (0.04–0.36) _a_	0.16 (0.03–1.87) _a_	0.11 (0.04–2.10) _a_
*Carcinogens*				
Ethanol (g/d)	2.18 (0.19–10.56)	1.88 (0.28–8.80) _a_	8.13 (1.76–22.93) _b_	6.02 (0.00–24.46) _a_
Xenobiotics				
B(a)P (µg/d)	0.06 (0.04–0.08)	0.06 (0.05–0.08) _a_	0.06 (0.04–0.08) _a_	0.07 (0.03–0.08) _a_
DiB(a)A (µg/d)	0.03 (0.01–0.10)	0.03 (0.00–0.04) _a_	0.05 (0.01–0.15) _b_	0.03 (0.00–0.32) _a_
Total PAH (µg/d)	1.09 (0.66–1.44)	0.93 (0.58–1.44) _a_	1.15 (0.75–1.43) _a_	1.22 (1.07–1.46) _a_
PhlP (ng/d)	82.56 (25.14–232.97)	77.78 (24.45–182.10) _a_	82.64 (23.79–329.91) _a_	83.12 (36.77–222.53) _a_
DiMelQx (ng/d)	6.67 (3.29–14.72)	5.13 (3.00–13.83) _a_	6.90 (3.47–16.83) _a_	9.96 (4.53–18.22) _a_
MelQx (ng/d)	23.50 (13.44–61.12)	22.15 (13.44–61.12) _a_	23.24 (13.42–56.27) _a_	25.27 (16.64–69.07) _a_
MelQ (ng/d)	0.81 (0.00–1.68)	0.93 (0.34–1.82) _a_	0.81 (0.00–1.30) _a_	0.00 (0.00–2.16) _a_
IQ (ng/d)	0.13 (0.00–0.27)	0.13 (0.00–0.25) _a_	0.13 (0.00–0.27) _a_	0.00 (0.00–0.17) _a_
Total HAs (sum) (ng/d)	119.54 (53.34–315.45)	103.17 (46.23–269.12) _a_	125.27 (53.95–381.99) _a_	185.87 (83.32–245.86) _a_
Nitrates (mg/d)	91.15 (55.92–140.60)	95.09 (65.41–140.60) _a_	69.44 (55.74–113.17) _a_	97.39 (54.94–186.96) _a_
Nitrites (mg/d)	2.39 (1.52–4.17)	2.48 (1.74–4.34) _a_	2.37 (1.31–4.17) _a_	2.44 (1.73–2.62) _a_
NDMA (µg/d)	0.16 (0.11–0.30)	0.16 (0.10–0.28) _a_	0.16 (0.11–0.30) _a_	0.17 (0.13–0.35) _a_
NPIP (µg/d)	0.07 (0.04–0.11)	0.08 (0.05–0.11) _a_	0.07 (0.03–0.11) _a_	0.06 (0.04–0.08) _a_
NPYR (µg/d)	0.11 (0.06–0.18)	0.12 (0.08–0.17) _a_	0.10 (0.05–0.18) _a_	0.09 (0.07–0.12) _a_
Acrylamide (µg/d)	14.70 (8.66–24.20)	15.07 (8.66–25.11) _a_	14.70 (8.06–21.42) _a_	14.15 (13.29–36.22) _a_

Values are presented as median (IQR = interquartile range = Q1–Q3). Values in the same row showing different subscripts display a statistically significant difference (*p* ≤ 0.05) from the control group. AαC (amino-alpha-carboline) and Comb. (combined nitroso compounds) are removed from the analysis due to extremely low frequency of consumption. B(a)P, benzo (a) pyrene; DiB(a)A, dibenzo (a) anthracene; Total PAHs, total polycyclic aromatic hydrocarbons; PhIP, 2-amino-1-methyl-6-phenylimidazo (4,5,b) pyridine; DiMeIQx, 2-amino-3,4,8 trimethylimidazo (4,5,f) quinoxaline; MeIQx, 2-amino-3,8 dimethylimidazo (4,5,f) quinoxaline; MeIQ, 2-amino-3,4 dimethylimidazo (4,5,f) quinoline; IQ, 2-amino-3-methylimidazo (4,5,f) quinoline; Total HA, total heterocyclic amines. NDMA, N-nitrosodimethylamine; NPIP, N-nitrosopiperidine; NPYR, N-nitrosopyrrolidine.

**Table 3:** GBD risk-related factors, bioactive and carcinogen tertiles as predictors of polyp risk.

	N (%)	Mean ± SD	OR (95% CI)	*p*-Value
GBD factors				
BMI				
5 kg/m^2^	93 (100)	27.24 ± 4.06	1.705 (0.975–2.980)	0.061
Alcoholic beverages (g/d)				
≤48.00	32 (37)	11.10 ± 14.11	–	–
>48.00	54 (63)	442.81 ± 559.93	2.539 (0.997–6.467)	0.051
Whole grains (g/d)				
≤50.00	78 (91)	5.47 ± 11.66	–	–
>50.00	8 (9)	130.69 ± 107.33	0.168 (0.029–0.966)	0.046 *
*Bioactives*				
Soluble pectin (g/d)				
≤0.57	32 (37)	0.43 ± 0.09	–	–
0.57–0.85	29 (34)	0.71 ± 0.08	0.357 (0.117–1.089)	0.070
≥0.85	25 (29)	1.33 ± 0.60	0.408 (0.125–1.327)	0.136
Flavonoids (mg/d)				
≤82.18	28 (33)	44.96 ± 22.29	–	–
82.18–251.34	30 (35)	152.20 ± 55.08	0.343 (0.112–1.052)	0.061
≥251.34	28 (33)	525.49 ± 323.63	1.099 (0.347–3.482)	0.872
Other polyphenols (mg/d)				
≤16.45	30 (35)	11.26 ± 4.56	–	–
16.45–32.15	28 (33)	23.91 ± 5.00	0.761 (0.249–2.324)	0.631
≥32.15	28 (33)	74.05 ± 52.77	0.358 (0.116–1.107)	0.074
*Carinogens*				
Ethanol (g/d)				
≤1.70	29 (34)	0.39 ± 0.56	–	–
1.70–11.62	28 (33)	5.33 ± 3.24	1.720 (0.575–5.148)	0.332
≥11.62	29 (34)	35.12 ± 25.93	3.542 (1.117–11.234)	0.032 *
DiB(a)A (µg/d)				
≤0.01	29 (34)	0.00 ± 0.00	–	–
0.01–0.07	28 (33)	0.04 ± 0.01	0.587 (0.191–1.803)	0.352
≥0.07	29 (34)	0.34 ± 0.33	3.100 (0.950–10.118)	0.061
Total PAH (µg/d)				
≤0.75	30 (35)	0.57 ± 0.14	–	–
0.75–1.29	27 (31)	1.07 ± 0.14	3.753 (1.154–12.204)	0.028 *
≥1.29	29 (34)	1.77 ± 0.37	1.530 (0.510–4.595)	0.448
Nitrates (mg/d)				
≤63.75	29 (34)	44.77 ± 13.95	–	–
63.75–106.65	28 (33)	85.25 ± 12.54	0.561 (0.182–1.729)	0.314
≥106.65	29 (34)	206.06 ± 102.67	0.371 (0.121–1.133)	0.082
Nitrites (mg/d)				
≤1.69	30 (35)	1.16 ± 0.38	–	–
1.69–3.34	26 (30)	2.45 ± 0.45	0.297 (0.094–0.944)	0.040 *
≥3.34	30 (35)	8.38 ± 13.07	0.515 (0.168–1.584)	0.247

The variables considered in this analysis were age, sex, risk of GBD-related factors (alcoholic beverages: 12, 24, 36, 48, 60, and 72 g/d; whole grains: 50, 100, and 150 g/d; milk: 60, 120, 180, and 240 g/d; red meat: 50, 100, 150, and 200 g/d; processed meat: 25, 50, 75, and 100 g/d; fiber: 10, 20, and 30 g/d; calcium: 300, 600, 900, and 1200 mg/d; physical activity: 2400, 3000, 3600, and 4200 METs/d; BMI: 5 kg/m^2^) and tertiles of consumption of all bioactives and carcinogens. Only variables showing significant (*) *p*-value < 0.05 or proximal (*p*-value < 0.10) results in at least one category are shown. For each variable considered, the lowest tertile is considered as the reference group. Values are adjusted for BMI and age. BMI, body mass index; CI, confidence interval; CRC, colorectal cancer; DiB(a)A, dibenzo (a) anthracene; GBD, Global Burden of Diseases, Injuries, and Risk Factors Study; MET; metabolic equivalent of task; OR, odds ratio.

## Data Availability

Not applicable.
